# Bacteriophage ZCKP1: A Potential Treatment for *Klebsiella pneumoniae* Isolated From Diabetic Foot Patients

**DOI:** 10.3389/fmicb.2018.02127

**Published:** 2018-09-11

**Authors:** Omar A. Taha, Phillippa L. Connerton, Ian F. Connerton, Ayman El-Shibiny

**Affiliations:** ^1^Biomedical Sciences, University of Science and Technology, Zewail City of Science and Technology, Giza, Egypt; ^2^Division of Food Sciences, School of Biosciences, University of Nottingham, Loughborough, United Kingdom; ^3^Faculty of Environmental Agricultural Sciences, Arish University, Arish, Egypt

**Keywords:** *Klebsiella*, bacteriophage, ulcer, diabetes, biofilm, osteomyelitis

## Abstract

The recorded growth in infection by multidrug resistant bacteria necessitates prompt efforts toward developing alternatives to antibiotics, such as bacteriophage therapy. Immuno-compromised patients with diabetes mellitus are particularly prone to foot infections by multidrug resistant *Klebsiella pneumoniae*, which may be compounded by chronic osteomyelitis. Bacteriophage ZCKP1, isolated from freshwater in Giza, Egypt, was tested *in vitro* to evaluate its lytic activity against a multidrug resistant *K. pneumoniae* KP/01, isolated from foot wound of a diabetic patient in Egypt. Characterization of ZCKP1 phage indicated that it belonged to the *Myoviridae* family of bacteriophages with a ds-DNA genome size of 150.9 kb. Bacteriophage ZCKP1 lysed a range of osteomyelitis pathogenic agents including *Klebsiella* spp., *Proteus* spp. and *E. coli* isolates. The bacteriophage reduced the bacterial counts of host bacteria by ≥2 log_10_ CFU/ml at 25°C, and demonstrated the ability to reduce bacterial counts and biofilm biomass (>50%) when applied at high multiplicity of infection (50 PFU/CFU). These characteristics make ZCKP1 phage of potential therapeutic value to treat *K. pneumoniae* and associated bacteria present in diabetic foot patients.

## Introduction

*Klebsiella* pneumoniae belongs to the *Enterobacteriaceae* family. It primarily affects patients with compromised defenses to cause severe complications. It is a particular problem for patients with diabetes mellitus leading to “diabetic foot” infections and osteomyelitis (Podschun and Ullmann, 1998). Once infection is established *K. pneumoniae* forms a biofilm that enables evasion of the host's defenses (Akers et al., 2014; Gupta et al., 2016). Moreover, phagocytosis by polymorphonuclear granulocytes is dramatically hindered, as *K. pneumoniae* possesses an outer protective polysaccharide capsule, a key determinant of their subsequent pathogenicity. The capsule suppresses complement components, particularly C3b (Domenico et al., 1994; Diago-Navarro et al., 2014). Among many other pathogenicity factors, bone adherence is attributed to adhesin production that may be fimbrial, or non-fimbrial (Malhotra et al., 2014). *Staphylococcus aureus* is considered the most frequently implicated bacterium in cases of diabetic foot infection (Richard, 2011) but recent data indicate that *K. pneumoniae* is responsible for approximately 21.7% of cases (Mukkunnath et al., 2015. With rising numbers of diabetes patients and the severity of foot osteomyelitis complications, this represents a considerable economic burden on health providers, notwithstanding the suffering of the individuals affected. In the past, *K. pneumoniae* was primarily associated with pulmonary and urinary infections, and was only relatively recently recognized as a significant cause of foot osteomyelitis (Dourakis et al., 2006; Prokesch et al., 2016).

Foot osteomyelitis is a common and serious problem in diabetic patients resulting chiefly from peripheral neuropathy or, less commonly, by vasculopathy and wound healing impediments (Grayson et al., 1995). It occurs in approximately two thirds of cases of diabetic foot patients (Grayson et al., 1995). *K. pneumoniae* is able to migrate to bone tissues haematogeneously (derived from or transported by blood) or contiguously from areas of local infections in the feet of diabetic patients (Mathews et al., 2010; Rana et al., 2013). If not effectively treated, viable cells of the infectious agent can be trapped in the devitalized bone and thus evade host defenses, and eventually cause chronic osteomyelitis (NADE, 1975; Ross et al., 2003; Calhoun and Manring, 2005).

In addition to the virulence characteristics described, the emergence of MDR *K. pneumoniae* strains, resistant to the last-line antibiotic treatment colistin, is a major concern (Kidd et al., 2017). Resistance arises from mutations of the *mgr*B gene, which are stably maintained in *Klebsiella* populations, from which resistance can be disseminated, in addition to plasmid mediated resistance due to mcr-1 and mcr-2 genes (Cannatelli et al., 2015). With the advent of the post-antibiotic era, severe cases of osteomyelitis may require more frequent surgical intervention in the form of resection of the infected and necrotic bone (Sanchez et al., 2013). It is therefore vital to seek alternative therapies to treat *K. pneumoniae* and other bacterial infections especially in developing countries (Nagel et al., 2016). Bacteriophage therapy is a good candidate and has been shown, using mice as animal models, to provide significant protection against respiratory and other infections caused by *K. pneumoniae* such as liver abscesses and bacteremia (Chhibber et al., 2008; Hung et al., 2011). Bacteriophage therapy has also been used to treat *K. pneumoniae* infected burn wound infections, in mice (Malik and Chhibber, 2009). Intranasal administration of lytic bacteriophage reduced the bacterial burden of *K*. *pneumoniae* in the lungs of mice (Cao et al., 2015). Other studies have characterized a number of diverse lytic bacteriophages to *K. pneumoniae* belonging to different families and demonstrated their potential *in vitro* (Bogovazova et al., 1991; Kesik-Szeloch et al., 2013; Hoyles et al., 2015). Bacteriophage therapy is regarded as a simple, safe and highly effective alternative to counter the rising problems associated with multidrug resistant bacteria (Qadir, 2015; El-Shibiny et al., 2017). Here we evaluate the lytic activity of bacteriophage ZCKP1 isolated from an environmental freshwater source in Egypt against a MDR *K. pneumoniae* KP/01 isolated from the foot of a diabetic patient.

## Materials and methods

### Bacterial strains and growth media

*K. pneumoniae* KP/01, used as a host for bacteriophage infection, was isolated from a human clinical diabetic-foot sample from a male patient in May 2016 and identified by National Institute of Diabetes using the VITEK method for identification (Cairo, Egypt). Other clinical isolates of *K. pneumoniae* (*n* = 21), *Proteus mirabilis* (*n* = 18) and *E. coli* (*n* = 15) were also isolated by National Institute of Diabetes, for bacteriophage host-range analysis, from wound infection samples and provided to the microbiology research lab at Zewail City. Isolates were kept in tryptone soy broth (TSB; Oxoid, England) containing (w/v) 20% of glycerol, at −80°C. In the following experiments, bacterial strains were grown on tryptic soy agar (TSA; Oxoid, England) overnight, and isolated colonies of bacteria were grown at 37°C, in TSB, to reach OD_600_ approximately 0.3.

### Bacterial identification using PCR specific primers and gel electrophoresis

PCR amplification was performed to confirm the identity of the *K. pneumoniae* isolate (KP/01) using specific primers for 16s RNA gene (forward primer: 5′-ATTTGAAGAGGTTGCAAACGAT-3′ and reverse primer: 5′-TTCACTCTGAAGTTTTCTTGTGTTC-3′; Woese and Fox, 1977; Woese et al., 1990). Thirty cycles were performed at denaturation temperature of 95°C for 30 s; annealing at 58°C for 60 s and extension at 72°C for 1 min looking for a PCR product of 133 bp length using an Applied Biosystems thermal cycler (Cady et al., 2012). The PCR product was run on a 1% (w/v) agarose gel to identify its size.

### Antibiotic sensitivity test

*K. pneumoniae* KP/01 strain was subjected to antibiotic resistance evaluation against a set of antibiotic discs including: tigecycline (TGC; 15 μg), imipenem (IPM; 10 μg), piperacillin-tazobactam (TZP; 100/10 μg), levofloxacin (LEV; 5 μg), linezolid (LZD; 30 μg), ceftazidime (CAZ; 30 μg), and cefepime (FEP; 30 μg) all from Oxoid (England). Antimicrobial sensitivity testing was performed for strains of *K. pneumoniae, E. coli* and *P. mirabilis*by using the disk diffusion methods in accordance with National Committee for Clinical Standards guidelines (Clinical and Laboratory Standards Institute, 1999). The antibiotics chosen are usually used for the treatment of diabetic foot infections in National Institute of Diabetes, due to their efficacy against members of the *Enterobacteriaceae*.

### Bacteriophage isolation, amplification and purification

Bacteriophages were isolated from environmental water samples from freshwater in El- Maryoteyya-Haram area, Giza, Egypt. *K. pneumoniae* (KP/01) used as a bacterial host upon which the clear plaquing phage were selected for further characterization. The bacteriophage plaques were purified by repeated single plaque isolation using sterile micropipette tips (Adams, 1959). All isolated bacteriophages were amplified in liquid culture (TSB) and the lysates were centrifuged at 6,400 × *g* for 15 min at 4°C to remove remaining bacterial cells and debris (Marcó et al., 2012). The supernatant containing phages was then centrifuged for 1 h 15,300 × *g* at 4°C. The pellet was resuspended in SM buffer (100 mM MgSO_4_.7 H_2_O; 10 mM NaCl; 50 mM TrisHCl; pH 7.5) and filtered using 0.22 μm syringe filters (Chromtech, Taiwan). Bacteriophage titers were determined using double-agar overlay plaque assays (Mazzocco et al., 2009).

### Examination of bacteriophage morphology by electron microscopy

The morphology of bacteriophage ZCKP1 was investigated using transmission electron microscopy at the National Research Center (Cairo, Egypt). Formvar carbon coated copper grids (Pelco International) were immersed into phage suspension, the phage were fixed using glutaraldehyde (2.5% v/v), washed and stained using 2% phosphotungstic acid (pH 7.0). After drying, grids were examined using a transmission electron microscope (JEOL 1230).

### Pulsed field gel electrophoresis (PFGE)

DNA was prepared from bacteriophage ZCKP1 (10^10^ PFU/ml) to determine the genome size by pulsed field gel electrophoresis (PFGE; Senczek et al., 2000). Briefly, bacteriophage suspended in agarose plugs were digested with lysis buffer (0.2% w/v SDS [Sigma]; 1% w/v N-Lauryl sarcosine [Sigma]; 100 mM EDTA; 1 mg/ml Proteinase K [Fischer Scientific]), overnight at 55°C. Following washing 2 mm slices of agaraose containing DNA were inserted into the wells of a 1% w/v agarose gel. The gel was run by using a Bio-Rad CHEF DRII system, in 0.5 X Tris-borate-EDTA, for 18 h at 6 V/cm with a switch time of 30 to 60 s. The size of the genome was determined by comparison to standard concatenated lambda DNA markers (Sigma Aldrich, Gillingham, UK).

### Phage DNA sequencing

Genomic DNA was prepared from phage ZCKP1 (10^10^ PFU/ml) lysates by proteinase K treatment (100 μg/ml in 10 mM EDTA pH 8) followed by resin purification using the Wizard DNA kit (Promega, UK) following the manufacturer's instructions. DNA sequencing was performed using the Illumina MiSeq platform. The data consisted of 3.1 million paired-end sequence reads of 250 bp in length. Initial processing of the raw data and *de novo* assembly was performed using CLC Genomics Workbench version 11.0.1 (Qiagen, Aarhus, Denmark). ORFs were predicted from PHASTER and manually curated (Arndt et al., 2016). Nucleotide sequences appear under the GenBank accession number MH252123.

### Lytic profiles of isolated bacteriophages

Using double-agar overlay plaque assays (Mazzocco et al., 2009), the lytic profile of phage ZCKP1 and other isolated phages was determined against a clinical isolate panel when spotted phage concentrations were not <10^9^ PFU/ml [34]. The experiment was performed using log phase bacteria. The panel included bacteria that cause osteomyelitis, including *K. pneumoniae, P. mirabilis* and *E. coli*. The lytic activity of bacteriophages was determined based on plaques of clear lysis. If ≥20 plaques were produced, the tested bacteria were regarded as being sensitive to the phages.

### Efficiency of plating

Bacteriophage ZCKP1 was tested in triplicate over eight decimal dilutions against all the susceptible bacterial strains lysed in the spot assays as previously described (Viazis et al., 2011). Conditions of these experiments were the same as spot test using log-phase bacteria. Thus, 200 μl of all bacterial isolates were added to top agar, and different dilutions of phages were spotted on petri dishes. The plates were incubated overnight at 37°C. Next day, EOP was estimated as the average PFU on target bacteria/average PFU on host bacteria.

### Determination of the frequency of bacteriophage insensitive mutants

The frequency of the emergence of bacteriophage insensitive mutants (BIMs) was estimated as previously described (O'Flynn et al., 2004). Phage ZCKP1 was mixed with bacterial host strains confirmed to be susceptible to the bacteriophage including strains of *K. pneumoniae, P. mirabilis*, and *E. coli* at an MOI of 100. After 10 min of incubation at 37°C, the suspension was serial diluted and spotted using double-agar overlay plaque assays. Plates were incubated overnight and BIM was calculated correspondingly by dividing bacterial viable counts remained after phage infection by initial viable counts. Experiments were conducted in triplicate.

### One step growth curve

One step growth curves were performed as previously described (Hyman and Abedon, 2009). Briefly, KP/01 strain was grown at concentration of 10^8^ and mixed with bacteriophage at multiplicity of infection of 1 and incubated at 37°C for 2 h. Directly after infection and every 10 min, aliquots of 200 μl were withdrawn and divided into two volumes of 100 μl. Chloroform was added to one of two volumes with a concentration of 1% (v/v); to set intracellular phages free while other 100 μl was left with no chloroform addition. After serial dilution, phage titer was estimated by spotting on top agar using double-layer method. Three replicates were conducted for each time interval.

### Bacteriophage potency against planktonic cells

The survival lysis characteristics of phage ZCKP1 were estimated KP/01 in the presence of ZCKP1 phage at multiplicities of infection of 0.1, 10 and 100 PFU/CFU was estimated in comparison to bacterial control at a temperature of 37°C (phage-free samples; Armon and Kott, 1993). Phage infective centers (IC) and plaque forming units (PFU) were also estimated, at different time intervals (0, 5, 10, 20, 30, 40, 60, 90, 120, and 180 min). IC is the amount of free phage particles released from the bacterial cells, without the need to add chloroform, while PFU refers to the number of nascent phage both inside and outside the bacterial cell. Briefly, two flasks were filled with either bacterial culture at a given concentration (control) or with bacterial culture at the same concentration and bacteriophage matching the desired MOI (Test). At every time interval, the concentration of bacterial control (B), bacterial survival (BS) IC, and PFU were simultaneously estimated. Bacterial concentration were determined using the Miles and Misra method (Miles et al., 1938), while phage concentration was estimated using double-agar overlay plaque assays by adding chloroform to the aliquot to be estimated in case of PFU determination, or not adding chloroform to calculate the IC.

Bacteriophage ZCKP1 was added to *K. pneumoniae* KP/01 in log-phase of growth, at 25°C, at an MOI of 1. Bacterial survival, number of infective centers, and number of plaque forming units were estimated periodically at different time intervals (0, 8, 24, 32, and 48 h).

### Bacteriophage activity against established biofilms of *K. pneumoniae*

The activity of ZCKP1 against established biofilms of KP1/01 was examined using a modification of previously described protocols (Cerca et al., 2005; Pettit et al., 2005). One hundred microliter aliquots of *K. pneumoniae* KP/01 (5 × 10^6^ CFU/ml) in 96-well flat-bottomed polystyrene microtitre plate (Sigma Aldrich) were incubated for 24 h at 37°C. Unattached planktonic cells were carefully removed. The number of bacterial cells in a biofilm per well were estimated to be 10^7^ CFU after 24 h (Mottola et al., 2013). Using different MOIs (5, 10, and 50), 100 μl aliquots of phage ZCKP1 diluted in TSB were added to each well, 1 day after biofilm establishment. Other wells received an equivalent amount of TSB as positive controls. In a parallel experiment, phage was introduced to wells every 4 h carefully replacing the previous suspension (containing TSB, planktonic cells and released phages) without disturbing the established biofilms. The biomass of preformed biofilms was quantified by staining with crystal violet (0.2% w/v). Following washing to remove excess dye with PBS, the crystal violet was solubilized in ethanol (95%). The absorbance was measured using a microplate reader at OD_600_ (Biotek, USA). The bacterial counts in biofilms were estimated using an MTT [3-(4,5-dimethylthiazol-2-yl)-2,5-diphenyltetrazolium bromide] assay (Serva Electrophores, Germany) as described by Cady et al. (2012). The absorbance was then measured at 570 nm at 4, 12, and 24 h, using a microplate reader (BioTek, USA). Control and test samples were assayed in triplicate.

### Bacteriophage pH and temperature stability

The temperature stability of phage ZCKP1 (10^10^ PFU/ml) was evaluated at 45, 55, 65, 75, 85, and 95°C, at 10 min intervals, over 1 h in adjusted water bath incubator. Immediately after incubation, serial dilutions of phage were spotted in triplicate, using standard double layer technique; on a lawn of host strain (KP/01) to estimate phage titers as previously described (Capra et al., 2004; Hammerl et al., 2014).

The bacterial counts of ZCKP1 at different pH values (5, 6, 7, 8, and 9) was determined after 1 h incubation, followed by determining the phage titer as previously described (Hammerl et al., 2014). Different pH values were achieved in SM phage buffer to maintain comparative conditions.

### Statistical analysis

In all data sets, test and control sets were compared using Student's *t*-test. A significance level of 0.05 was applied in all cases. Analytical statistics were undertaken using GraphPad PRISM version 7.00 for Windows (GraphPad Software, La Jolla, USA).

## Results

### *Klebsiella* identification and sensitivity to antibiotics

The identity of the KP/01 strain was confirmed to be *K. pneumoniae* by PCR, by the presence of 133 bp band corresponding to conserved region in 16s RNA gene of *K. pneumoniae*, following amplification with the specific primers. The antibiotic sensitivity of *K. pneumoniae* isolate KP/01 was tested using the disc diffusion method and the results showed that *K. pneumoniae* isolate KP/01 was sensitive to tigecycline (TGC), imipenem (IPM) and piperacillin-tazobactam (TZP) but resistant to levofloxacin (LEV), linezolid (LZD), ceftazidime (CAZ) and cefepime (FEP).

### Bacteriophage isolation

Bacteriophages were isolated from freshwater near the pyramids of Egypt in Giza. Selection of the bacteriophage was undertaken upon serial passage according to their ability to lyse a broad range of *K. pneumoniae* isolates and other pathogens causing osteomyelitis, generate reproducible clear zones of lysis, produce hallow zones around lysis zones indicative of exopolysaccharide depolymerase activity and capable of replication to produce high titers on the selected host with respect to time. Bacteriophage ZCKP1 fulfilled these criteria.

### Morphology of lytic ZCKP1 phage

**Figure 1:**
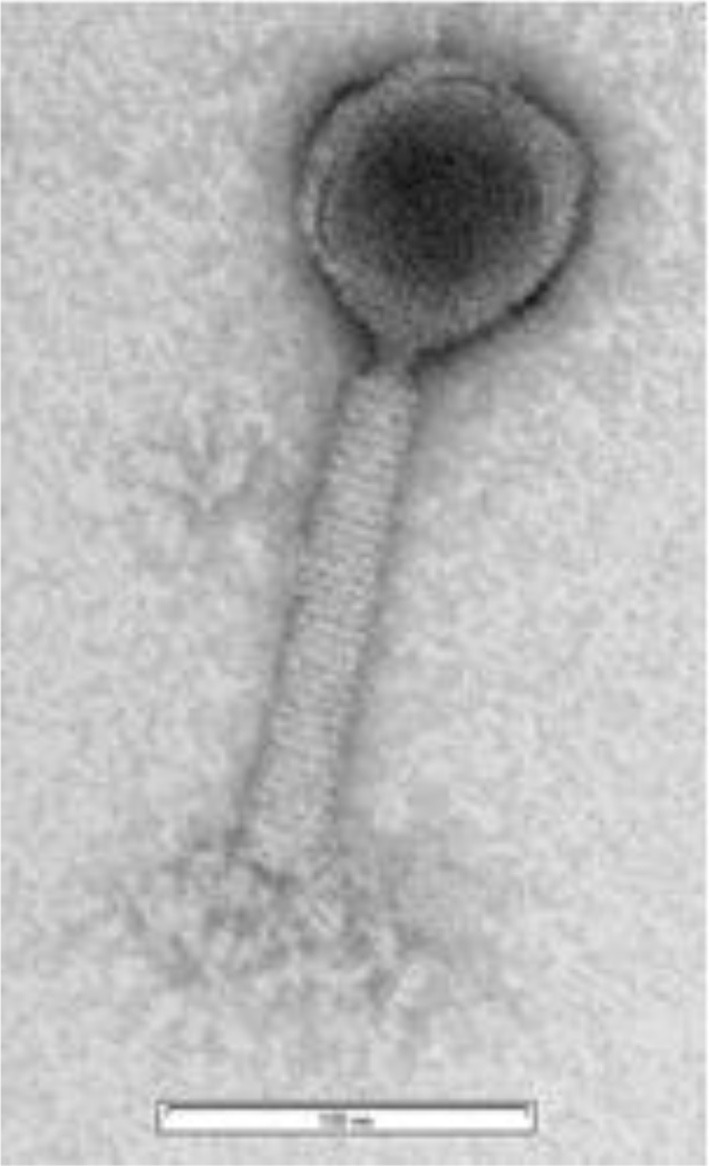
Transmission electronmicroscopic image of phage ZCKP1.

Electron microscopy revealed that ZCKP1 had an icosahedral head and contractile tail with collar, and base plate, and therefore typical of phages belonging to the family of *Myoviridae* (Figure 1). The proportions of the phage head and tail length were also typical of the *Myoviridae* with the head size being 80 ± 0.7 nm while tail length was calculated to be 138.5 ± 2.5 nm.

### Phage genome

Bacteriophage ZCKP1 contains a double-stranded DNA genome estimated to be 160 kbp by PFGE, which is comparable to values indicated by International Committee on Taxonomy of Viruses (ICTV) for bacteriophages belonging to the *Myoviridae* family. DNA sequencing of the phage DNA enabled *de novo* assembly and accurate size determination of a circular permuted genome of 150,925 bp with a G + C content of 39.1%. The genome contained 267 open reading frames, the majority of which are hypothetical proteins or recognized in BLASTP database searches as phage proteins without any ascribed function. Reading frames for which putative functional information could be ascribed to the products appear in Supplementary Table 1. Notably these include the phage structural proteins, nucleotide metabolism and components of the replication machinery that are conserved amongst *Myoviridae* infecting hosts within the *Enterobacteriaceae*. Of interest are enzymes that have the potential to modify infected cell surface polysaccharides that may impede superinfection. These include an O-antigen biosynthesis protein, a glycosyltransferase and a wcaM superfamily protein associated with colonic acid biosynthesis clusters present in *Enterobacteriaceae* that feature exopolysaccharide production. Four genes encoding proteins related to tellurite resistance are present. Tellurite resistance is frequently used for selection in culture isolation media but is not used for antimicrobial therapy. The genes are thought to contribute to colicin and phage resistance (Taylor and Summers, 1979), which may provide reasons for their presence in phage ZCKP1 in that colicin resistance will provide a selective advantage to the phage infected cell and phage resistance to prevent superinfection. Also of note the phage encodes a member of the hydrolase 2 superfamily implicated in bacterial cell wall hydrolysis. The nearest database phage sequence was PHAGE_Escher_phAPEC8 that infects avian pathogenic *E. coli* and is also a member of the *Myoviridae* (Tsonos et al., 2012).

### Bacteriophage host range and efficiency of plating

The host range of five different phages isolated from freshwater, including phage ZCKP1 were tested on bacteria that were isolated from diabetic patients suffering from osteomyelitis. The ZCKP1 phage was capable of producing lysis zones (≥20 plaques) on 15 out of 21 *K. pneumoniae* isolates, 5 out of 18 *P. mirabilis* isolates and 9 out of 30 *E. coli* isolates, while other phages did not display a comparable spectrum of activity against the *K. pneumoniae* isolates (Table 1). A range of EOP for ZCKP1 phage was observed against different species of *Enterobacteriacae* (Supplementary Table 2). For *K. pneumoniae* seven phages demonstrated EOPs similar to the multidrug resistant host strain. For *P. mirabilis*, all susceptible strains showed EOP <0.1, whereas for *E. coli six* strains supported replication with EOPs approaching that of the permissive *K. pneumoniae* hosts (Table 2).

**Table 1:** Lytic activity of isolated phages against *K. pneumoniae* and other selected members of the *Enterobacteriaceae*.

**Bacteriophage name**	**Bacteriophage activity**				
	**ZCKP1**	**P2**	**K4**	**EC4**	**P9**
*K. pneumonia* (21 isolates)	15	3	5	1	2
*P. mirabilis* (18 isolates)	5	5	0	0	8
*E. coli* (30 isolates)	9	0	0	2	0

**Table 2:** Efficiency of plating of phage ZCKP1 against different species of *Enterobacteriacae*.

**Bacterial species**	***K. pneumoniae* (*n* = 15)**	***P. mirabilis* (*n* = 5)**	***E. coli* (*n* = 9)**
EOP >0.5	7	–	6
EOP >0.1 < 0.5	6	–	2
EOP >0.001 < 0.1	1	5	1

### Frequency of BIMS

BIMs were recovered following high multiplicity infections (100) of host bacteria *K. pneumoniae, P. mirabilis* and *E. coli* with bacteriophage ZCKP1 at 37°C. Mutational frequencies of 7.5 × 10^−5^ ± 1.7 × 10^−4^ and 3.7 × 10^−5^ ± 6.8 × 10^−5^ were determined for *Klebsiella* and *E. coli*, respectively where *K. pneumoniae* KP1 alone exhibited a lower frequency of 5 × 10^−6^ ± 4.04 × 10^−6^.

### *In vitro* characterization of phage ZCKP1

**Figure 2:**
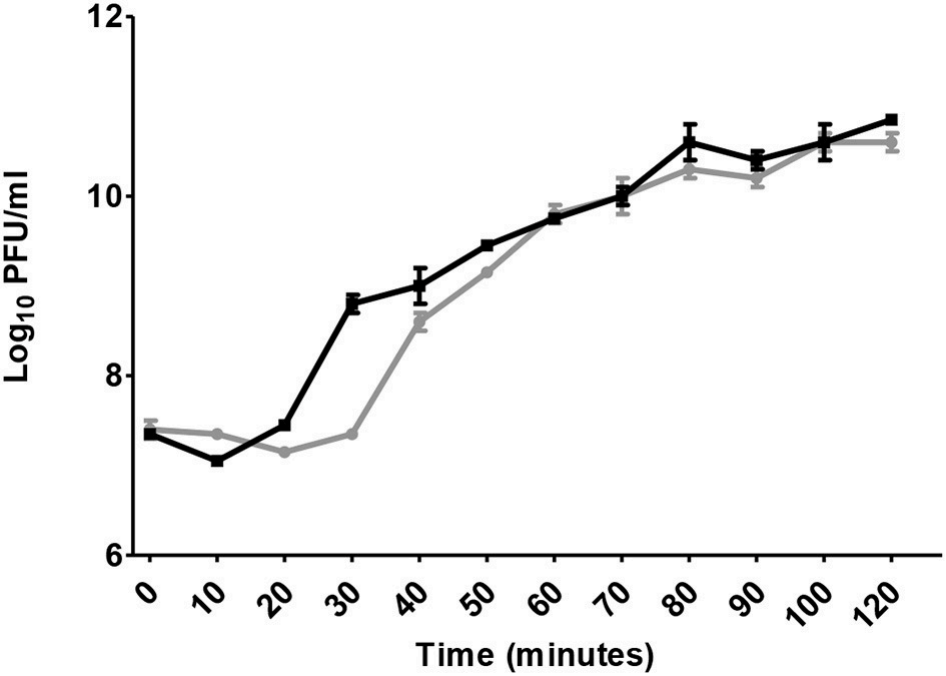
Single step growth curve. Gray dashed line represents nascent phage without chloroform addition (PFU/ml), while the black line represents phages released after chloroform addition (PFU/ml).

A single-step growth curved demonstrated bacteriophage virions were naturally released from bacterial cells after 30 min: the latent period which is the time taken for phages to be assembled and released after infection. However, viruses were assembled 10 min before. This was indicated by eclipse period that was estimated to be 20 min, as chloroform aids new phage particles to free from bacterial cell wall (Figure 2). Burst size was estimated to be ~110 virions per single bacterium.

**Figure 3:**
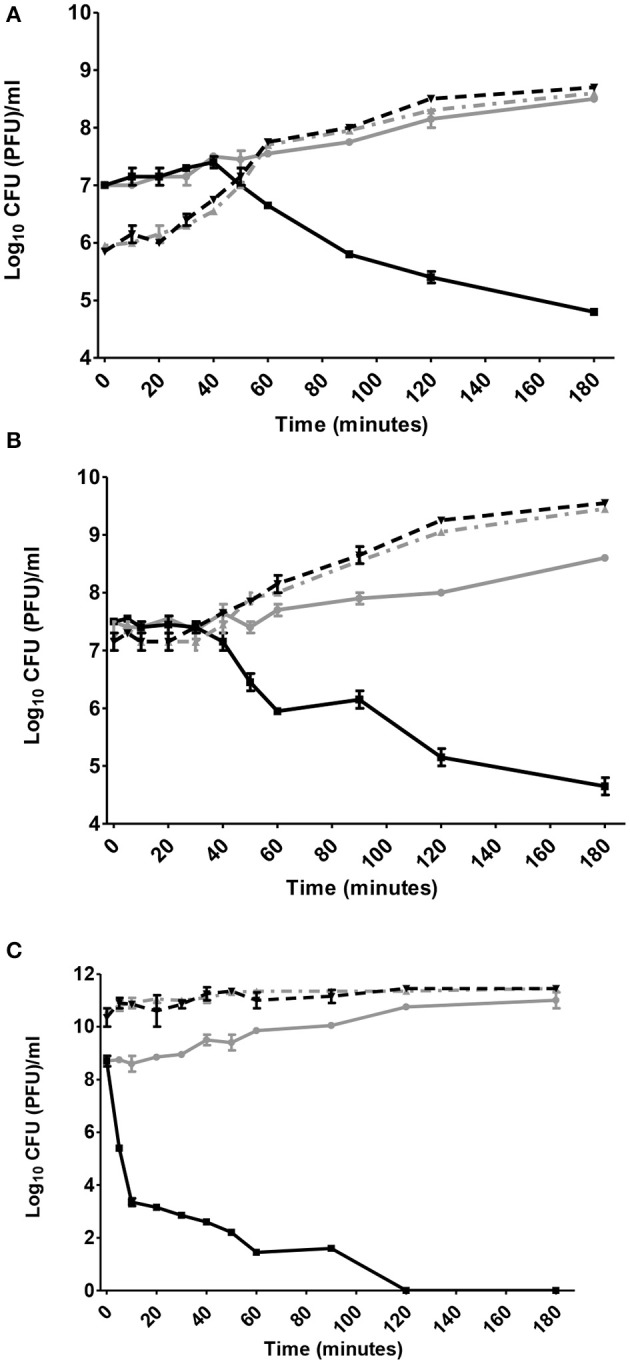
*In vitro* activity of phage ZCKP1 at 37°C. Panels show bacterial counts and phage titers of *K. pneumoniae* KP/01 infected with ZCKP1 at: **(A)** MOI 0.1; **(B)** MOI 1; **(C)** MOI 100. Black solid line represents viable count of *K. pneumoniae* KP/01 infected with phage (CFU/ml); Gray solid line represents *K. pneumoniae* KP/01 uninfected control (CFU/ml); black dashed line represents phage infective centers (PFU/ml) and gray dashed line represents nascent phage (PFU/ml).

The infection and lysis characteristics of phage ZCKP1 were estimated at different MOIs, over a period of 3 h (Figures 3A–C) in a growing culture of *K. pneumoniae* KP/01 (Figures 3A–C). *K. pneumoniae* KP/01 was lysed by phage ZCKP1 at each MOI tested but the MOI of 100 reduced the viable bacteria from 9.0 log_10_ CFU/ml to below the limit of detection at 37°C by 2 h (Figures 3A–C). Under these circumstances the reductions in bacterial count were not accompanied by a measurable rise in phage titer (Figure 3C). Phage replication was observed at lower MOI, which coincided with the commencement of the fall in viable count.

### Bacteriophage activity against *K. pneumoniae* established in biofilms

**Figure 4:**
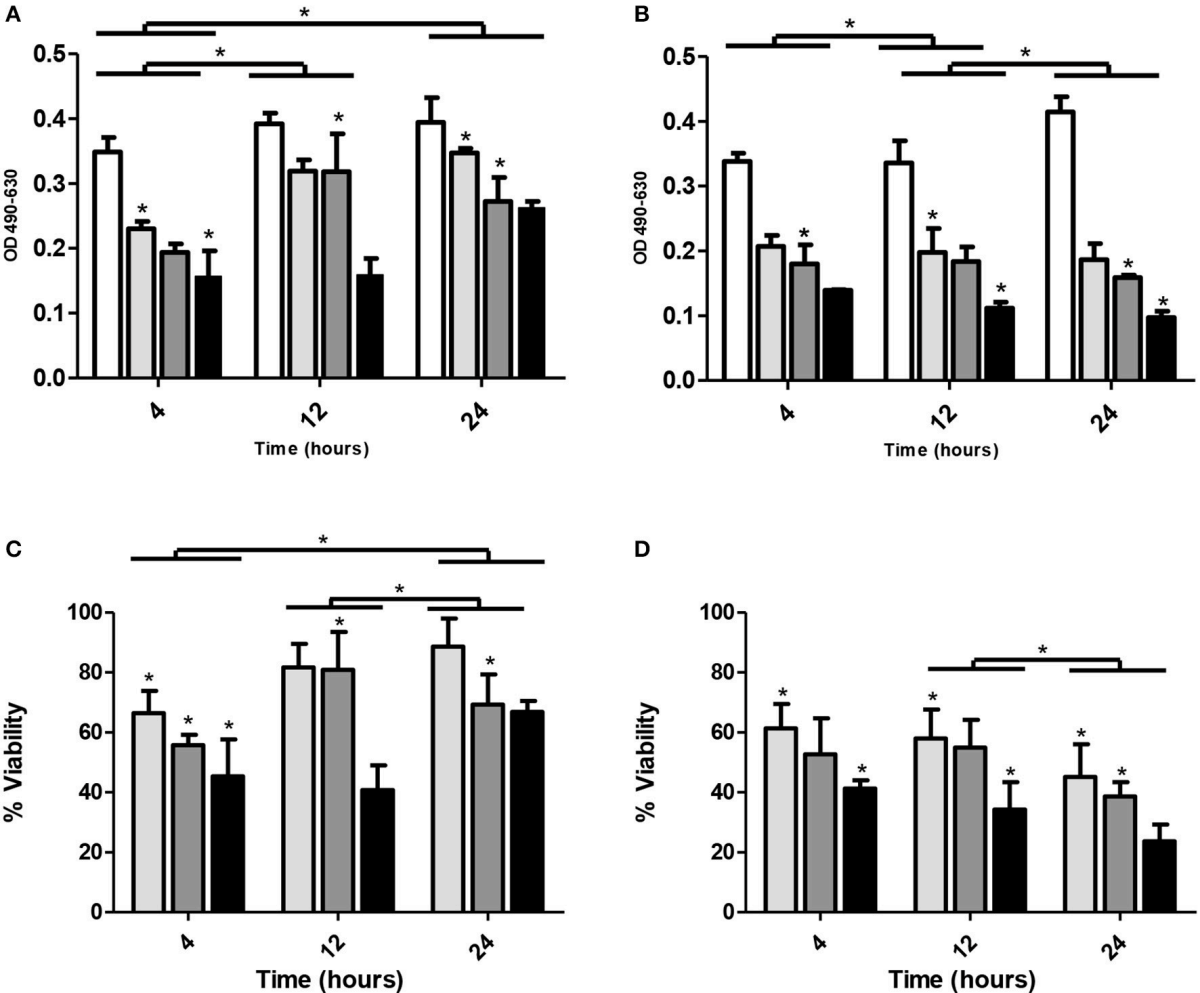
Phage treatments of *K. pneumoniae* KP/01 biofilms. Panels **(A)** and **(B)** show the effect of phage treatment on preformed biofilms determined by crystal violet staining and solubilization estimates of biomass: **(A)** single treatment with phage ZCKP1; **(B)** with multiple treatments with phage ZCKP1 using different MOIs. White columns represent untreated control; light gray columns represent a starting MOI of 5; dark gray columns represent a starting MOI of 10 and solid black columns represent a starting MOI of 50. Panels **(C)** and **(D)** show bacterial counts in biofilms determined using an MTT assay, **(C)** single treatment with phage ZCKP1 bacteriophage or **(D)** with multiple treatments with phage ZCKP1 bacteriophage using different MOIs: Light gray columns represent a starting MOI of 50; dark gray columns represent a starting MOI of 10 and solid black columns represent a starting MOI of 5. *P < 0.01 (brackets specify comparisons between groups).

A single application of ZCKP1 to established biofilms of *K. pneumoniae* KP/01 resulted in a reduction crystal violet stainable biofilm content (*P* < 0.01; Figure 4A) and the percentage of viable cells observed by MTT staining (*P* < 0.01; Figure 4C) after 4 h. The most effective treat represented the highest MOI (50 PFU/CFU). However, following this disruption there was recovery in biofilm estimates accompanied by a recovery in cell viability. Multiple treatments of phage ZCKP1 on established *K. pneumoniae* KP/01 biofilms at 4 h intervals resulted in significant reductions in biofilm content and prevented the recovery of cell viability throughout the 24 h period of the experiment (*P* < 0.01; Figures 4B,D).

### Bacteriophage temperature and pH stability

**Figure 5:**
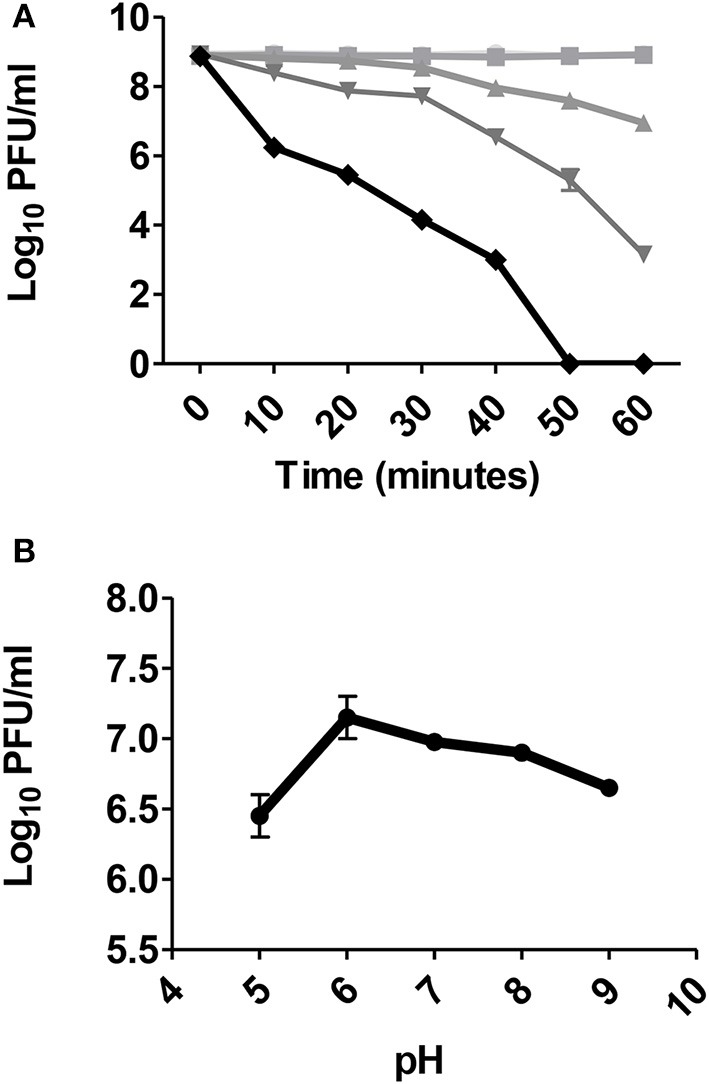
The stability of phage ZCKP1 at **(A)** different temperatures (45°C: circle, 55°C: square, 65°C: triangle, 75°C: inverted triangle and 85°C: diamond) and **(B)** pH values. Results are shown as means ± Standard error.

The stability of phage ZCKP1 at different temperatures and pH values was investigated (Figures 5A,B). Phage titers were stable, at approximately 10^9^ PFU/ml, for 1 h at temperatures of 45 and 55°C. The phage titer decreased after 40 min at 65°C to 10^8^ PFU/ml, and continued to decline below 10^7^ PFU/ml after 1 h. A significant decline (*P* < 0.005) was observed when phages were incubated at 75 and 85°C. However, phage could still be recovered after 1 h at 75°C at a titer of 10^3^ PFU/ml. Phage could not be recovered after 40 min at 85°C. Acidic pH of < 6 significantly (*P* < 0.005) reduced the phage stability after 1 h. The optimum stability was observed to be pH 6 but persisted at alkaline pH values to pH 9 (Figure 5B).

## Discussion

*K. pneumoniae* is an enteric pathogen that causes pneumonia and wound infections (Podschun and Ullmann, 1998). The effectiveness of antibiotics to treat such infections has been reduced significantly in recent years due to the increasing numbers of antibiotic-resistant bacteria, and as a result morbidity and mortality remain high. Antibiotic resistance is a growing public health threat for which the use of bacteriophage as an alternative to antibiotics may be considered to combat MDR infections. In particular phage therapy has also been considered a promising approach to eliminate diabetic foot ulcer after infection by MRSA in human subjects (Fish et al., 2016). In order to get the maximum benefits of bacteriophage based therapies, it is important to determine the characteristics of individual bacteriophages so that treatments can be tailored for the situation where treatment is to be applied. Moreover, it is crucial to ensure that phages selected do not have the capacity to transfer resistance or pathogenic traits to the resident microbiota (Abedon and Thomas-Abedon, 2010).

Antibiotics that were previously effective in the elimination of diabetic foot infections are now less effective. The *K. pneumoniae* KP/01 isolate recorded here shows resistance to levofloxacin, fluoroquinolone and was identified as a ceftazidime-resistant *K. pneumoniae* (CSKP). Ceftazidime is a cephalosporin antibiotic that can be degraded by extended spectrum beta lactamases (ESBL) that include SHV, TEM, CTX and YOU types (Sougakoff et al., 1988; Urban et al., 1994). *K. pneumoniae* KP/01 also showed resistance to the cephalosporin cefepime. As a clinical multiple drug resistant bacteria the KP/01 isolate was an ideal host for this study (Sougakoff et al., 1988).

Both morphological analysis and genome size confirmed that bacteriophage ZCKP1 belonged to the *Caudovirales* order with typical features of *Myoviridae*. It had an icosahedral head, a contractile tail with base plates showing tail fibers and spikes in addition to a collar. The genome of 151 kb, differs in size to the 45 kbp of KLPN1 phage previously reported as isolated against *K. pneumoniae* (Hoyles et al., 2015). Bacteriophage ZCKP1 demonstrated a broad lytic profile covering a variety of bacterial pathogens including *K. pneumoniae, Proteus* and *E. coli* that all contribute to osteomyelitis cases and were isolated from patients with “diabetic foot.”

*In vitro* studies of potential therapeutic bacteriophages ensures only the most effective phages progress to clinical trials based on their capability to lyse pathogens in planktonic and biofilm formations with wide host range coverage. Phage ZCKP1 was shown to be highly effective at reducing *K. pneumoniae* counts *in vitro* and proved to be stable at high temperatures and over a wide pH range. Phage ZCKP1 was also effective against other members of *Enterobacteriacae* that cause osteomyelitis, which contributes to the therapeutic potential. With the application of high concentrations of bacteriophages (MOI of 100), ZCKP1 was demonstrated to reduce *K. pneumoniae* without producing new phages. This is an established phenomenon called “lysis from without,” where many phages become absorbed to bacterial cells causing lysis without release of new phage (Abedon, 2011). In addition, a single high dose applied in a clinical situation may enable the human immune system to overcome reduced numbers of pathogens by working synergistically with the phage. Even with lower doses of phage, the rate of development of resistance to bacteriophages is approximately 10-fold lower than the rate of the development of antibiotic resistance (Carlton, 1999). The conditions of application and the influence of immune system can vary so the action of a particular phage must be considered before therapeutic use (O'Flynn et al., 2004; Lu and Koeris, 2011). In this context the mutation frequencies determined at high MOI applications would dictate the use of phage cocktails, and possibly the availability of reserve phage. Developing a cocktail of isolated lytic phages may increase the efficacy of bacteriophages to lyse multiple hosts and reduce the frequency that resistant strains may emerge.

*Klebsiella* are able to form thick biofilms on tissues and on medical implants making them more resistant than free-living planktonic cells to antibacterial agents and have reduced susceptibility to antibiotics (Calhoun and Manring, 2005). Phage ZCKP1 treatment of *K. pneumoniae* KP/01 biofilms was shown to be an effective method for biofilm reduction, although repeated treatments were required to prevent regrowth. Reductions in biofilm biomass have been attributed to the action of a soluble exopolysaccharide depolymerase (Cornelissen et al., 2011). These enzymes have the ability to disrupt the capsule of *Klebsiella* making it more susceptible to antibacterial agents (Hughes et al., 1998; Kesik-Szeloch et al., 2013). The nucleotide sequence of phage ZCKP1 revealed enzyme activities consistent with polysaccharide modification, However, the presence of wcaM could influence exopolysaccharide structure to adversely affect biofilm integrity when embedded bacteria become phage infected.

Previously reported phage treatments of *K. pneumoniae* biofilms include: a phage belonging to the *Podoviridae* family (Chhibber et al., 2013); a *Siphoviridae* named bacteriophage Z (Jamal et al., 2015) and *Myoviridae* phages (Kesik-Szeloch et al., 2013). Of these, the Myoviridae are likely the most promising as they represent virulent bacteriophage that do not mobilize and transfer genetic information. The gene sequence of phage ZCKP1 suggests that it does indeed fall into this category. Four genes associated with tellurite resistance were observed but are not used for antimicrobial therapy. Tellurite resistance is often associated with colicin and phage resistance phenotypes (Taylor and Summers, 1979), and likely extends this advantage to the virus infected cell as insurance against superinfection.

## Conclusion

Phage ZCKP1 has been fully characterized *in vitro* and shows excellent potential to be used as a therapeutic agent against *K. pneumoniae* infections of diabetic foot. It can reduce the bacterial pathogen in both planktonic and biofilms and is extremely stable over a range of pH and temperatures. Therapeutic trials are needed to confirm its potential *in vivo*.

## Data availability

All data generated or analyzed during this study are included in this published article and are available from the corresponding author. Nucleotide sequences appear in the NCBI public database under the GenBank accession number MH252123.

## Author contributions

AE-S: primary responsibility for design of the work. OT and AE-S: substantial contributions to the design of the work and analysis and interpretation of the data. OT, PC, IC, and AE-S: drafting the work and revising it critically for important intellectual content. OT, PC, IC, and AE-S: final approval of the version to be published.

### Conflict of interest statement

The authors declare that the research was conducted in the absence of any commercial or financial relationships that could be construed as a potential conflict of interest.

## Abbreviations

BIMs: bacteriophage insensitive mutants
IC: phage infective centers
MOI: multiplicity of infection
MTT, 3-(4, 5-dimethylthiazol-2-yl)-2: 5-diphenyltetrazolium bromide.
