# Supplementary material for: Bacteriophage ZCKP1: A Potential Treatment for Klebsiella pneumoniae Isolated From Diabetic Foot Patients
Source: Front Microbiol. 2018 Sep 11;9:2127. doi: 10.3389/fmicb.2018.02127 (PMC6141743; doi:10.3389/fmicb.2018.02127)
Supplement: Supplementary file 1 [file Table_1.docx]

Supplementary Tables:

Supplementary Table 1: Phage encoded products with putative functions

| **CDS Location** | **Putative Product** |
| --- | --- |
| complement(3249..4049) | NAD-dependent protein deacetylase of SIR2 family |
| complement(9969..10712) | Serine/threonine protein phosphatase (EC 3.1.3.16) |
| complement(14531..15289) | Phosphate starvation-inducible protein PhoH, predicted ATPase |
| complement(15327..15827) | T4-like phage baseplate hub & tail lysozyme |
| complement(16250..17092) | dTDP-4-dehydrorhamnose reductase (EC 1.1.1.133) |
| complement(17086..17652) | dTDP-4-dehydrorhamnose 3,5-epimerase (EC 5.1.3.13) |
| complement(18487..19572) | Ribonucleotide reductase of class Ia (aerobic), beta subunit (EC 1.17.4.1) |
| complement(20106..22349) | Ribonucleotide reductase of class Ia (aerobic), alpha subunit (EC 1.17.4.1) |
| complement(26230..27189) | Phage exonuclease |
| complement(28599..29477) | DNA recombination-dependent growth factor C |
| complement(30505..31620) | DNA ligase, phage-associated |
| complement(32780..33712) | RNA ligase, phage-associated |
| complement(33714..34265) | Phosphohydrolase (MutT/nudix family protein) |
| complement(34265..34621) | Phage anti-restriction nuclease |
| 40185..40526 | HigA protein (antitoxin to HigB) |
| 42970..45045 | Phage terminase, large subunit |
| 48843..49844 | elements of external origin; phage-related functions |
| 60781..61254 | Enterobacterial phage protein JK_75P |
| 61254..64130 | Phage tail fibers |
| 64262..66292 | Phage capsid and scaffold |
| 66301..68538 | Phage tail fibers |
| 68858..69016 | Phage tail length tape-measure protein |
| 72932..73477 | Phage tail fibers |
| 73826..76879 | Colanic acid biosynthesis protein wcaM |
| 76944..78968 | Phage tail fiber protein |
| 79010..81676 | Fibronectin type III domain protein |
| complement(84782..88114) | T7-like phage DNA Polymerase (EC 2.7.7.7) |
| complement(88114..88908) | putative type II methylase protein( EC:2.1.1.-) |
| complement(89442..91220) | DNA primase/helicase, phage-associated |
| complement(91511..92455) | glycosyltransferase |
| complement(92463..93410) | O-antigen biosynthesis protein |
| complement(95978..96580) | Tellurium resistance protein TerD |
| complement(97091..97885) | Tellurium resistance protein TerD |
| complement(99281..100393) | Tellurite resistance protein |
| complement(102606..103580) | Integral membrane protein TerC |
| complement(106688..107158) | Ribonucleotide reductase of class III (anaerobic), activating protein (EC 1.97.1.4) |
| complement(107155..108969) | Ribonucleotide reductase of class III (anaerobic), large subunit (EC 1.17.4.2) |
| complement(111219..112811) | putative DNA helicase |
| 123673..124215 | COG3236 / GTP cyclohydrolase II (EC 3.5.4.25) |
| 146672..147343 | Resolvase, N-terminal |
