# Supplementary material for: Bacteriophage ZCKP1: A Potential Treatment for Klebsiella pneumoniae Isolated From Diabetic Foot Patients
Source: Front Microbiol. 2018 Sep 11;9:2127. doi: 10.3389/fmicb.2018.02127 (PMC6141743; doi:10.3389/fmicb.2018.02127)
Supplement: Supplementary file 2 [file Table_2.docx]

Supplementary Table 2: Efficiency of plating.

| **#** | **Bacterial isolate** | **EOP** |
| --- | --- | --- |
| *Klebsiella* | | |
| 1 | K3 | 0.333333 |
| 2 | K5 | 0.666667 |
| 3 | K6 | 0.5 |
| 4 | K8 | 0.333333 |
| 5 | K9 | 1.666667 |
| 6 | K11 | 1.666667 |
| 7 | K13 | 0.333333 |
| 8 | K15 | 0.833333 |
| 9 | K16 | 0.033333 |
| 10 | K17 | 0.166667 |
| 11 | K18 | 1.833333 |
| 12 | K19 | 1.866667 |
| 13 | K20 | 0.333333 |
| 14 | K21 | 0.333333 |
| *Proteus* | | |
| 1 | P1 | 0.001333 |
| 2 | P6 | 0.013333 |
| 3 | P7 | 0.006667 |
| 4 | P11 | 0.001333 |
| 5 | P15 | 0.002333 |
| *E. coli* | | |
| 1 | EC7 | 6.666667 |
| 2 | EC17 | 1.333333 |
| 3 | EC19 | 1 |
| 4 | EC21 | 2.333333 |
| 5 | EC23 | 0.033333 |
| 6 | EC25 | 0.006667 |
| 7 | EC27 | 0.333333 |
| 8 | EC28 | 0.666667 |
| 9 | EC30 | 0.03 |
